# Surface-Coated Acupuncture Needles as Solid-Phase Microextraction Probes for In Vivo Analysis of Bioactive Molecules in Living Plants by Mass Spectrometry

**DOI:** 10.3390/metabo13020220

**Published:** 2023-02-02

**Authors:** Huiyun Cheng, Xu Zhao, Lin Zhang, Mingying Ma, Xiaoxiao Ma

**Affiliations:** 1 Medical College of Qinghai University, Xining 810001, China; 2 Department of Chemistry, Tsinghua University, Beijing 100084, China; 3 State Key Laboratory of NBC Protection for Civilian, Beijing 102205, China; 4 Department of Precision Instrument, Tsinghua University, Beijing 100084, China

**Keywords:** surface-coated acupuncture needles, solid-phase microextraction, nano-electrospray mass spectrometry

## Abstract

In this work, we report the coupling of solid-phase microextraction (SPME) enabled by surface-coated acupuncture needles with nano-electrospray mass spectrometry (nanoESI-MS) for the analysis of bioactive molecules in living plants. The needle tip was oxidized by a mixture of nitric acid and hydrogen peroxide solution and then subject to surface coating via carbonization of paraffin. A combination of oxidation and surface coating resulted in a thin coating of carbon film, whereby the significantly increased surface area promoted both analyte enrichment and ionization for MS analysis. The analytical performances were evaluated through the characterization of small molecules, peptides and proteins. Compared with conventional nanoESI, our new strategy of employing surface-coated needles had a high salt tolerance. The streamlined experimental workflow could be completed within one minute. The linear dynamic ranges for L-histidine and L-lysine, as two representatives, were over two orders of magnitude with a limit of detection (LOD) of 3.0~5.0 ng/mL. A mark is made on the needle at 2 mm from the tip, the needle is then kept in the sample for 30 s. In vivo sampling and identification of *α*-tomatine and organic acids from the stem of a living tomato plant were demonstrated as a practical application, while the physiological activities of the plant were not disrupted due to the minimally invasive sampling. We anticipate that the developed strategy may be of potential use for real-time clinical and other on-site analyses.

## 1. Introduction

Mass spectrometry (MS) is a powerful tool in chemical, biological and biomedical research, and has found wide application in clinical diagnosis, environmental monitoring and food safety [1]. Inaugurated by Fenn et al. in 1989 [2,3], electrospray ionization (ESI)-MS has become the method of choice for the analysis of biomolecules, and can be innately coupled with liquid chromatography for separation and sensitive MS analysis [4]. In 1994, Mann et al. [5,6] developed the nano-electrospray ionization (nanoESI) technique using a pulled glass capillary (1~3 μm at the tip) as the electrospray emitter. In nanoESI, the need for forced flow and nebulizing gas was eliminated, and the required volume of sample solution was greatly reduced to 10~50 nL/min, in contrast with the typical flow rate of 1000 nL/min in conventional ESI. However, both ESI and nanoESI requires that the target analytes be extracted from the sample to form a solution, therefore sample pretreatment is critical for analytical performance. To address this critical issue, solid-phase microextraction (SPME), pioneered by Pawliszyn et al. [7], has become a popular choice. Based on the solid-phase extraction (SPE) technique, SPME integrates sampling, extraction and introduction of analytes of interest, which enables versatile, fast and non-exhaustive analysis of samples of minimal amount. SPME has been widely used in the areas of chemical, biological, pharmaceutical and environmental analysis [8]. Increasing focus has been put on the coupling of novel SPME techniques with mass spectrometry to achieve fast, confident and multiplexed analysis of target analytes, as demonstrated in the coupling of SPME with nanoESI [9] and ambient ion sources including desorption electrospray ionization (DESI) [10,11,12], direct analysis in real time (DART) [13], desorption corona beam ionization (DCBI) [14,15] and coated-blade spray (CBS) [16], to name just a few. Promising results have been obtained which demonstrate the capability of SPME probes in the extraction and enrichment of analytes for MS identification and quantitation.

Specifically, probe electrospray ionization-mass spectrometry (probe ESI-MS) [17,18] is another method that is similar in principle with SPME-MS, aiming to achieve in situ and in vivo MS analysis. The metal needle used for sampling was also used to generate the electrospray for sample analysis after application of a high voltage. Based on probe ESI-MS, Zhang et al. reported the MS detection of metabolites at the cellular and subcellular levels using tungsten needles for analyte enrichment [19]. They also demonstrated the coupling of a TiO_2_-coated SPME probe with nanoESI-MS for the enhanced detection of phosphopeptides [20].

Acupuncture has a long history of being used in traditional Chinese medicine to treat various types of diseases. For instance, pain could be relieved through the insertion and manipulation of acupuncture needles at specific points (acupoints) of the human body [21]. In recent years, acupuncture has become increasingly popular in the United States and worldwide. The basic tools of acupuncture, thin needles made of stainless steel, are disposable. As the needle is inserted and then pulled out of the body, a small amount of tissue is sampled onto the needle tip, similar to a biopsy. Considering these features, it was expected that by extending the probe ESI concept to acupuncture needles, a new method that allowed easy sampling and sensitive MS detection could be readily developed and easily implemented, in particular for the rapid in vivo detection of plant metabolites [22,23,24,25,26,27].

Herein, we demonstrate the coupling of a carbon film-coated acupuncture needle, for use as an SPME probe, with nanoESI-MS for the enrichment and detection of bioactive metabolites in living plants. A combination of oxidation and surface coating resulted in a thin carbon film coating where the increased surface area aided both analyte enrichment and ionization for MS analysis. For sample analysis, the needle was first inserted into a region of interest for analyte extraction and enrichment. After that, the needle was placed into a pulled nanoESI capillary preloaded with electrospray solvent for analyte elution. Electrospray was generated by applying a high voltage on the other end of the needle. This technique was capable of analyzing small-molecule compounds, peptides and proteins. The rapid analysis of an extract from the stem of a living tomato plant revealed abundant *α*-tomatine, malic acid and citric acid, among a large number of metabolites. The whole experimental process was completed within one minute, without damaging or disrupting the physiological activities of the plant. This method is simple in terms of needle fabrication and operation and offers improved MS detection sensitivity. It is not only useful in the analysis of living plants, but can potentially be used to analyze living animals in such aspects as therapeutic drug monitoring (TPM) and the real-time analysis of disease biomarkers.

## 2. Materials and Methods

### 2.1. Reagents and Materials

Methanol (HPLC grade) was purchased from Mreda Technology Inc. (Beijing, China). Nitric acid (95%) and hydrogen peroxide (30%) were purchased from Beijing Chemical Works (Beijing, China). Salicylic acid, L-lysine, L-histidine, isotope-labelled L-histidine, L-arginine, L-carnosine, cytochrome c and angiotensin II were purchased from Beijing Biodee Biotechnology Co., Ltd. (Beijing, China). All reagents were analytical pure and used as received. Deionized water was prepared from a Milli-Q water purification system (Billerica, MA, USA). Except for L-histidine and L-lysine, other standard substances are not subject to quantitative analysis, and are therefore they are not presented in Table 1.

Acupuncture needles (0.40 × 60 mm) were purchased from Suzhou Medical Appliance Factory (Suzhou, China). Helium (99.999%) and nitrogen (99.999%) were purchased from Huayuan Gas (Beijing, China). Tomato seeds bought from local markets were sown on a humidified, unconsolidated growth soil. Room temperature was kept at 28 °C with a relative humidity of 60%, and the photoperiod was maintained at 16 h. Two weeks later, the seedlings were transplanted to a 2 L pot filled with the same growth soil and were irrigated three times a week.

Borosilicate glass capillaries (0.59 mm i. d., 1.0 mm o. d.) were purchased from VitalSense Scientific Instruments Co., Ltd. The capillaries were pulled using a P-2000 micropipette puller (Sutter Instrument, Novato, CA, USA) preprogrammed to fabricate nanoESI emitters with an inner diameter of 3 μm at the tip. Parameters of the instrument were set as follows: Heat = 340, Fil = 5, Vel = 28, Del = 128, and Pull = 60.

### 2.2. Mass Spectrometry

MS measurements were carried out on a Finnigan LTQ ion trap mass spectrometer (Thermo Scientific, San Jose, CA, USA). The commercial ionization source was removed and replaced with a homemade nanoESI ionization source. For tandem MS analysis, helium was used as the collision gas, the normalized collision energy was 35% and the isolation width of *m*/*z* was set at 1.0. Other parameters of the mass spectrometer were set as follows: capillary temperature = 275 °C, tube lens voltage = 50 V, maximum inject time = 50 ms, microscans = 1. Peak height was used for quantitation and a typical 30 s interval was used for averaging the spectrum.

### 2.3. Fabrication of Surface-Coated Acupuncture Needles

The fabrication of oxidized acupuncture needles was simple and straightforward. The tip of an acupuncture needle was first dipped in a mixture of nitric acid (95%) and hydrogen peroxide (30%) (75/25, *v*/*v*) solution for 20 min. Oxidation took place as evidenced by bubbles generated on the metal needle surface. The modified tip of the needle was then coated with paraffin wax and placed in a flame of 400–500 °C until it had burned red for 30 s. Cooled to room temperature, the needle was washed by water, alcohol and water, successively. The needle was finally dried under nitrogen and stored for subsequent SMPE and MS analysis. Scanning electron microscope (SEM) analysis was performed on an SU-8000 instrument (Hitachi, Tokyo, Japan) prior to and after such a surface modification.

### 2.4. SPME Via Surface-Coated Needles Coupled with NanoESI-MS Analysis

**Figure 1:**
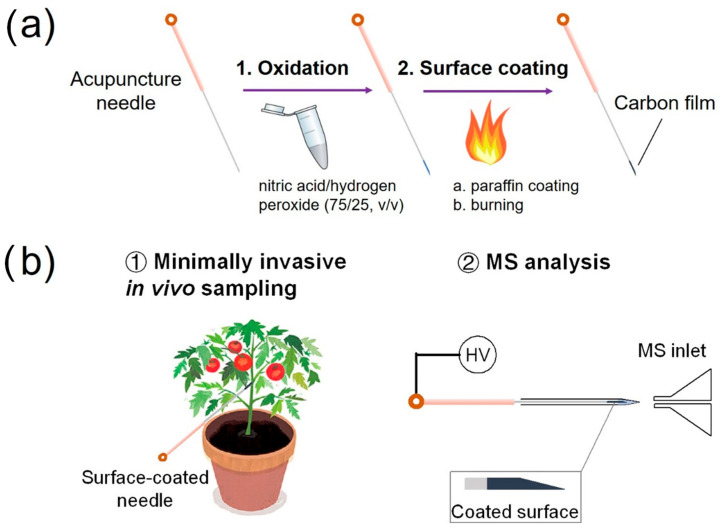
(**a**) Scheme for preparing a surface-coated acupuncture needle. (**b**) Experimental workflow for using a surface-coated needle for the analysis of a living plant.

Details for the preparation of the surface-coated acupuncture needles and the experimental workflow of sample analysis via SPME using the needle (2~5 mm) coupled with nanoESI-MS analysis are shown in Figure 1a,b. For analyte sampling in a solution, the tip of the needle was immersed in the solution for 30 s, taken out and then inserted from the back opening of a nanoESI emitter preloaded with 500 nL methanol/water (50/50, *v*/*v*). The tip was in direct contact with the solution. In such a relatively large volume of samples, it is reasonable to speculate that the solution concentration might remain constant during SPME. Therefore, the dependence on extraction volume can be neglected. As to the surface coating, no displacement was observed to occur, as evidenced by the clean mass spectrum after SPME. Following extraction, a high voltage of 2.3 kV was applied to the needle to initiate the electrospray. The distance between the needle tip and the MS inlet was 5 mm.

## 3. Results and Discussion

### 3.1. Characterization of the Surface-Coated Acupuncture Needles

**Figure 2:**
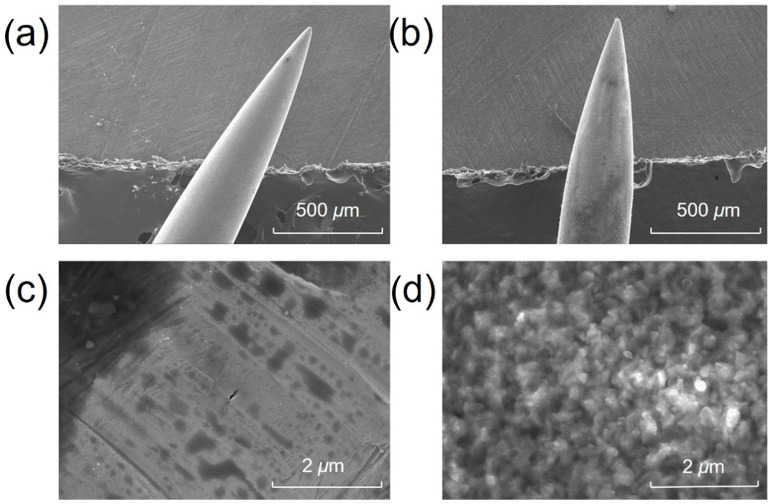
Scanning electron microscope (SEM) images of the acupuncture needle tip (**a**,**c**) prior to and (**b**,**d**) after surface oxidation.

The surface morphology and elemental composition of the acupuncture needle tips were investigated prior to and after surface coating. Figure 2 presents the SEM images of the needle tip prior to and after surface modification. The surface of the untreated needle tip was smooth, as demonstrated in Figure 2a,c. After surface coating, it became rough and porous (Figure 2b,d), suggesting a significantly increased surface area. Energy-dispersive spectroscopy (EDS) analyses revealed that the compositions of Fe, Cr, Ni, C, and O on the surface of untreated needle tip were 67.0%, 18.4%, 7.6%, 5.8% and 0.8%, respectively. By contrast, the compositions of Fe, Cr and Ni on the surface of treated needle tip decreased to 56.2%, 17.1% and 6.2%, respectively, while the composition of oxygen increased from 0.8% to 8.5% due to the oxidation process. The coating method was selected because it is strongly bounded to the needle, in addition to its high specific surface area and ease of operation. The composition of carbon was 11.6%, twice that of an untreated tip.

### 3.2. Analytical Figures of Merit

**Figure 3:**
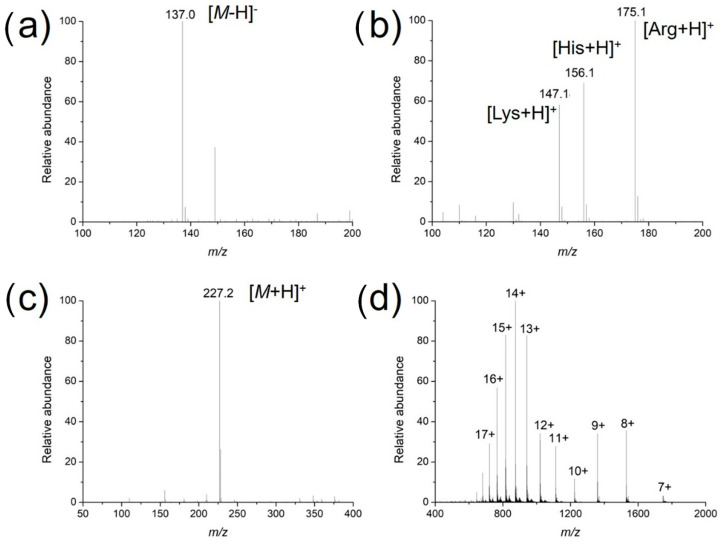
Mass spectra of different analytes ionized via surface-modified acupuncture needles: (**a**) An amount of 1 μM salicylic acid in the negative ion mode; (**b**) a mixture of L-lysine, L-histidine and L-arginine at equimolar concentrations of 1 μM; (**c**) 1 μM L-carnosine; and (**d**) 10 μM cytochrome c in the positive ion mode.

**Figure 4:**
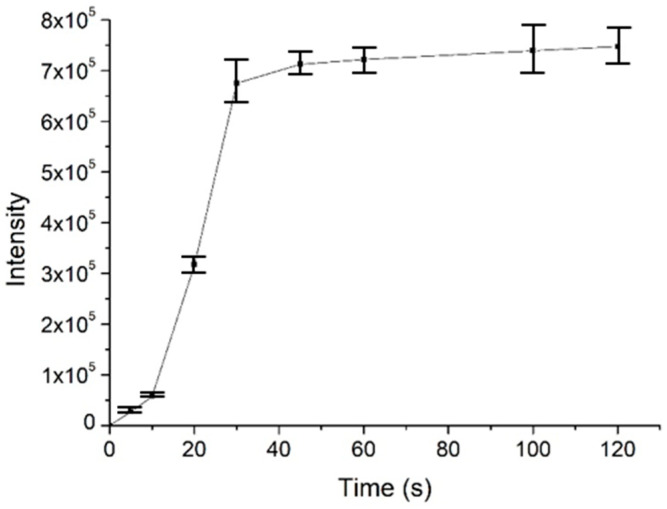
The enrichment performance of the surface-coated needle tip as an SPME probe, using angiotensin II as an example. The experiments were repeated three times (*n* = 3).

To investigate the usability, stability and enrichment performances of the surface-coated acupuncture needle coupled with nanoESI-MS, a number of analytes, including small molecules, peptides and proteins were selected for analysis. Take salicylic acid as the example, the needle (2~5 mm) was immersed in 1 μM salicylic acid solution for 30 s for enrichment, followed by elution in the nanoESI emitter and MS analysis. As demonstrated in Figure 3a, abundant MS signals of protonated salicylic acid (*m*/*z* 137.00) were detected with high signal-to-noise ratios. Following sample collection, upon deposition of 0.5 μL solution on the needle tip, ion signals could last for ~50 s. Other candidate analytes, including amino acids (a mixture of L-lysine, L-histidine and L-arginine), L-carnosine and cytochrome c were detected in a similar way (Figure 3b–d). The enrichment performance of the needle tip was also evaluated, using 2 μM angiotension II as an example. The signal intensity of the analyte was plotted against the time of extraction. As demonstrated in Figure 4, the intensity increased rapidly in the first 30 s, after which it reached a plateau. Therefore, all analytes were enriched for 30 s prior to MS analysis. Since the purpose of this work is pre-concentration and detection, as long as saturation of the tip does not affect sensitive MS detection, we were not overly concerned if the needle coating will saturate under high concentrations. Enrichment is observed for most analytes using the fiber for the same concentration, by 3~10 folds.

The quantitative analytical performances of the developed method were investigated using L-histidine and L-lysine. A series of standard solutions containing L-histidine and isotope-labelled L-histidine (internal standard, IS) were prepared to plot calibration curves. The concentration of IS was kept constant at 500 ng/mL while the concentrations of L-histidine varied. The intensity ratio of L-histidine to the IS was plotted against the concentration ratio of L-histidine to the IS. Good linearity was obtained for the calibration curve. The linear range of L-histidine was 50–5000 ng/mL, and the limit of detection (LOD) was 5.0 ng/mL. Similar evaluations of L-lysine gave a linear range of 10–1000 ng/mL, and the LOD was determined to be 3.0 ng/mL. The LODs were calculated based on a concentration with a signal-to-noise ratio of three. The LODs were comparable to those obtained using nanoESI-MS, but the present method was faster and simple. For reproducibility study, one probe was used for six consecutive extractions of 500 ng/mL L-histidine or L-lysine spiked in water, and the relative standard deviation (RSD) of the signal intensities was below 5%. A probe-to-probe repeatability study was carried out by analyzing 500 ng/mL L-histidine or L-lysine using six different probes, with the RSDs below 8.0%. The recoveries of analytes ranged from 97.5% to 105.6%. Together, these results suggest that the strategy of coupling the probe for analyte enrichment with nanoESI-MS was reliable and reproducible. It is believed that internal standard (IS) introduction will benefit quantitation, by improving reproducibility and detection accuracy. Without IS, the relative standard deviation (RSD) of the measured concentration is ~30%. The repeatability is ~25%, in contrast with the values of 2.4~7.6% shown in Table 1. The LODs are comparable in both cases with or without IS, while the linearity of the regression equations are significantly improved. For in vivo analysis, IS introduction is difficult, so we were required use the relative intensities of analytes of interests for relative quantitation.

### 3.3. Comparison of Needle-Tip Extraction/NanoESI with Conventional NanoESI

**Figure 5:**
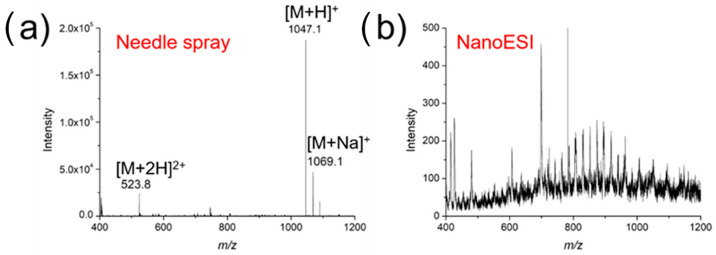
Mass spectra of angiotensin II (10 μM, with 1% NaCl) (**a**) using a surface-coated needle for nanoESI and (**b**) conventional nanoESI in the positive ion mode.

One notable advantage of using the surface-coated needle for SMPE was its high salt tolerance, which was demonstrated in the analysis of a 10 μM angiotensin II solution containing 1% NaCl. As shown in Figure 5a, singly and doubly protonated angiotensin II were clearly observed using nanoESI-MS after SPME, with little interference from salt. In the case of conventional nanoESI-MS analysis, however, no peaks corresponding to angiotensin II could be detected, while MS signals of salt clusters dominated the spectrum (Figure 5b). This clearly suggests the high salt tolerance is as a result of the highly selective peptide extraction and enrichment. It is worth noting that direct in vivo analysis without pre-concentration is not quite compatible enough with nESI to enable highly sensitive MS analysis.

### 3.4. Identification of Bioactive Molecules in Living Plants

**Figure 6:**
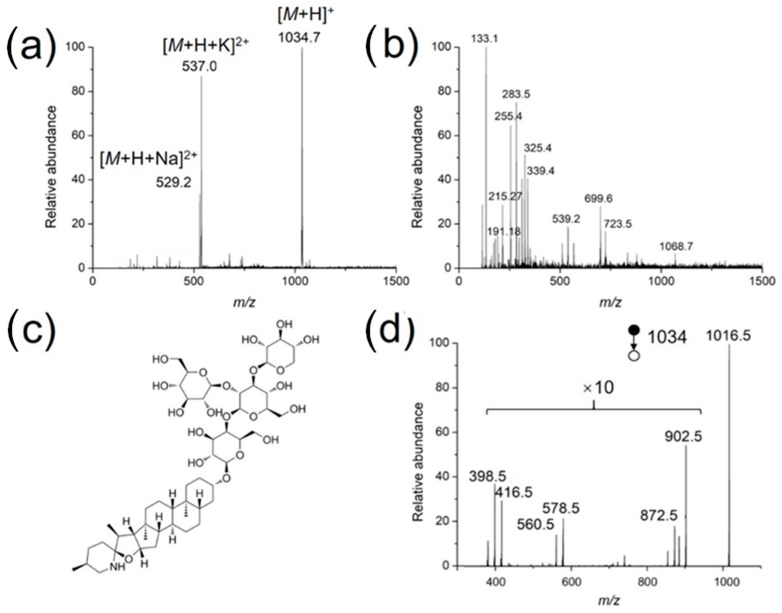
Full scan mass spectra of the extract from the stem of a living tomato in the (**a**) positive and (**b**) negative ion modes, (**c**) structure of *α*-tomatine, and (**d**) tandem mass spectrum of protonated *α*-tomatine.

As a practical application, the developed method was used to identify bioactive compounds in the stem of a living tomato plant. The coated needle was inserted directly into the stem for sampling (2 mm), followed by MS analysis in a nanoESI emitter preloaded with 500 nL methanol/water (50/50, *v*/*v*). As mentioned before, for low concertation samples, a long extraction time is needed to achieve better detection sensitivity. For both the standard solutions and tomato samples, an extraction time of 30 s works fine with respect to the detection of target compounds. In the positive ion mode, *α*-tomatine was clearly detected, as doubly charged sodium adduct ([M + H + Na]^2+^), doubly charged potassium adduct ([M + H + K]^2+^) and protonated form ([M + H]^+^) were detected with high abundances at *m*/*z* 529.27, 537.00 and 1034.73, respectively (Figure 6a). A series of phytochemicals were detected in negative ion mode (Figure 6b), assigned as organic acids, such as malic acid (*m*/*z* 133.18), or citric acid (*m*/*z* 191.18). Figure 6d demonstrates the MS/MS spectrum of protonated *α*-tomatine, which is in accordance with that reported by the leaf spray MS method [28]. Protonated α-tomatine is not detected, because a high collision energy (CE) was used, *m*/*z* 1016 is the fragment after water loss from protonated α-tomatine (*m*/*z* 1034).

## 4. Conclusions

In this study, we have developed a novel method employing surface-coated acupuncture needles for rapid in vivo sampling and analyte enrichment, followed by nanoESI-MS analysis. High-quality full scan and tandem mass spectra were acquired due to analyte enrichment, with improved salt tolerance and detection sensitivity comparable to conventional nanoESI-MS. This method was used for the sensitive analysis of small-molecule compounds, peptides and proteins. As a practical application, it was applied for the in vivo sampling and identification of *α*-tomatine and organic acids from the stem of a living tomato plant. The presented strategy should find wide applications in clinical diagnosis and in vivo biological monitoring. It can also be readily integrated with a field-portable mass spectrometer to enable highly sensitive and rapid in situ and in vivo analyses.

## Figures and Tables

**Table 1:** Quantitative analysis of L-histidine and L-lysine.

Analyte	Linear Range (ng/mL)	Regression Equation	R^2^	LOD (ng/mL)	Repeatability (%)		Accuracy (Mean ± SD), %, *n* = 3	
					One Tip	Tip-Tip	50 ng/mL	500 ng/mL
L-histidine	50–5000	y = 0.4218x + 0.0402	0.9991	3.0	3.4	7.6	97.5 ± 5.3	105.6 ± 5.8
L-lysine	10–1000	y = 2.092x + 0.0371	0.9996	5.0	2.4	4.8	98.2 ± 2.2	103.5 ± 5.7

## Data Availability

The data presented in this study are available in the main article.
